# SARS-CoV-2: a new threat

**DOI:** 10.1515/almed-2020-0083

**Published:** 2020-11-02

**Authors:** Cristina A. López Rodríguez, Marc Boigues Pons, Bibiana Quirant Sánchez, Aina Teniente Serra, Joan Climent Martí, Eva Ma Martínez Cáceres

**Affiliations:** Immunology Division, Laboratori Clínic Metropolitana Nord, Germans Trias i Pujol University Hospital, Barcelona, Spain

**Keywords:** COVID-19, immune response, pandemic, SARS-COV-2

## Abstract

**Background:**

The pandemic caused by the emergence of the new SARS-CoV-2 virus worldwide has had a major impact at all levels and has forced in-depth research into its behavior, pathogenicity and treatment.

**Content:**

This review provides an overview of various aspects of the virus and the immune response it triggers, as well as a description of the different diagnostic and therapeutic approaches adopted.

**Summary:**

SARS-COV-2 is a RNA virus with some peculiarities that make it different from its predecessors SARS-CoV and MERS. Given its structural characteristics and pathogenesis, it can cause different clinical manifestations as the disease progresses. The immune system has been proven to play a major role in the response to this virus and, therefore, the study of antibodies and lymphocyte populations during the different stages of the disease is crucial.

**Outlook:**

The knowledge of the effect of the virus and the immune response is crucial for the development of good quality vaccines, therapies and diagnostic techniques, which are essential for the control and eradication of the disease.

## Introduction

### SARS-CoV-2

By the end of December 2019, there were reports of cases of pneumonia of unknown origin whose focal point was the wholesale seafood and animal market in Wuhan, in the province of Hubei, China [1]. After an outbreak of cases, samples of bronchoalveolar fluid (BAF) of patients with confirmed and suspected pneumonia were analyzed. The purpose of these analyses was to study the genetic material and obtain the genomic sequence of the infectious agent. Finally, a novel coronavirus, SARS-CoV-2, was detected and identified as a β-coronavirus. This novel coronavirus shared some characteristics with other viruses that caused epidemics in the past, such as SARS and MERS, but with some peculiarities [1].

### Characteristics of the novel SARS-CoV-2

Coronavirus (CoV) belongs to the family *Coronaviridae,* which are enveloped, single-stranded RNA viruses with a genome of 26–32 kilobases in length [2].

These viruses cause a large variety of respiratory, enteric, hepatic and neurological diseases. There are four subfamilies of coronavirus: α, β, γ and δ-CoV. Human infections by CoV are caused by α and β-CoV. Other β-CoV, such as SARS-CoV and MERS-CoV, induce severe life-threatening infections [3].

**Figure 1:**
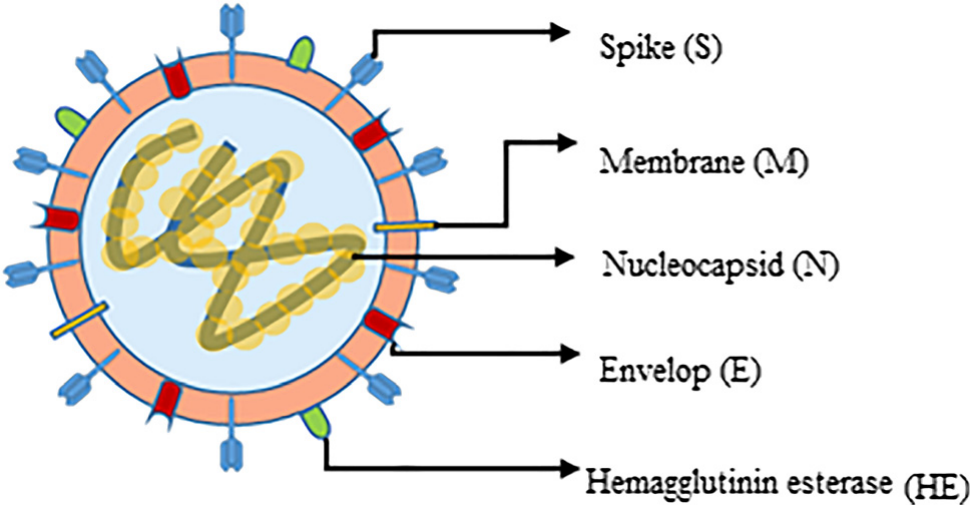
Structure of SARS CoV-2. Adapted from Jin y et al*.* [3].

The structure of SARS-COV-2 is similar to that of other β-CoVs: The virion presents a nucleocapsid composed of RNA and the phosphorylated nucleocapsid protein (N). This nucleocapsid is enveloped by a double layer of phospholipids that contain two spikes: the trimeric spike glycoprotein (S) and hemagglutinin esterase (HE) [3]. HE serves as a receptor-destroying enzyme to facilitate the release of the virion from infected cells and escape from attachment to nonpermissive host cells [4]. Protein S is a viral fusion protein that promotes the attachment of viral and cellular membranes during entry. Protein S is also the main target of neutralizing antibodies elicited during infection and one of the targets of vaccine design [4].The membrane protein (M) and the envelope (E) protein are located in the viral envelope [3] (Figure 1).

### Pathogenesis

As SARS-CoV, SARS-CoV-2 uses ACE2 receptor (angiotensin converting enzyme-2) to enter the host [5]. This type of receptor is located in the lungs, heart, kidneys and gut [3]. ACE2 is associated with cardiovascular diseases such as hypertension, where these receptors are overexpressed by a compensatory mechanism of the disease [6].

The virion attaches to receptor ACE2 through glycoprotein S, which contains two subunits, S1 and S2. Subunit S1 determines cell tropism by the receptor-binding domain (RBD), whereas S2 mediates viral cell membrane fusion induced by two tandem domains (HR1 and HR2) [7].

After binding to the receptor, protein S undergoes a conformational change that facilitates fusion of the viral envelope with the cell membrane by the endosomal pathway. Then, SARS-COV-2 releases RNA into the host cell. RNA is translated into viral replicase polyproteins pp1a and pp1ab, which are then cleaved into small products by viral proteases. The polymerase produces a series of subgenomic mRNAs by discontinuous transcription and finally is translated into relevant viral proteins. Viral proteins and genomic RNA are subsequently assembled into virions in the ER and Golgi and then transported by vesicles and released out of the cell [8].

### Stages of infection and clinical characteristics

Several authors, as Siddiqi et al. describe three clinical stages of the disease [9].

**Stage I**: Early establishment of the disease: This stage involves an incubation period of 1–14 days, generally 3–7 days associated with mild and often nonspecific symptoms such as malaise, fever and dry cough [7]. In this stage, SARS-COV-2 multiplies and establishes residence in the host, primarily focusing on the respiratory system. During the incubation period, the patient can transmit the disease through respiratory droplets and secretions and by direct contact [7]. The most frequent laboratory alterations are elevated C-reactive protein (CRP), hepatic enzymes and mild lymphopenia and neutrophilia [9].

**Stage II (moderate)**: Pulmonary involvement (IIa) without and (IIb) with hypoxia: During the second week, up to 80% of patients develop viral pneumonia, with cough, shortness of breath, fever and hypoxia (defined as a PaO_2_/FiO_2_ of <300 mmHg). Radiological findings include bilateral infiltrates or opacities in frosted glass both on chest X-ray and on computerized axial tomography. Blood analysis reveals lymphopenia and transaminitis. Systemic inflammation markers [IL-6, erythrocyte sedimentation rate (ESR), PCR, D-dimer and ferritin] are elevated, although not excessively [9].

**Stage III (severe)**: Systemic hyperinflammation: This is the most severe stage of the disease, which is reached by approximately 15% of patients. This stage manifests as an extra pulmonary systemic hyper-inflammation syndrome. In this stage, systemic inflammation markers (including IL-6) are very elevated and may simulate a hemophagocytic lymphohistiocytosis (SHLH) syndrome. Critical patients exhibit elevated levels of proinflammatory cytokines, D-dimer, ferritin, troponins and N-terminal prohormone of brain natriuretic peptide (NT-proBNP), with marked neutrophilia and lymphopenia. These patients can also develop shock, vasoplegia, respiratory failure and even cardiopulmonary collapse. Finally, the disease can cause multiorganic failure [9].

## Immune response

### Cellular response

Immune response in patients with COVID-19 is divided into three stages, which overlap with the clinical stages described above:

(1) Viremia stage. In this stage, viral RNA in blood increases dramatically during the first week. Initial innate response involves the production of type-I interferons (IFN)-I in the airways. This stage is crucial for viral replication control and the activation of an effective immune cell response [10].
(2) Acute or pneumonia phase. This stage occurs at 7–10 days from the onset of infection. This stage is characterized by inflammation with neutrophilia, increased proinflammatory cytokines and chemokines (interleukin (IL) −6, tumor necrosis factor (TNF)-⍺, IFN-I and -II, IL-8, CXCL-10); decrease in T and B lymphocytes in peripheral blood; and alterations in several biochemical and hematological parameters, such as an increase in D-dimer, ferritin, lactate dehydrogenase (LDH), CRP, and a decrease in albumin [11]. During this stage, the viral load reaches its maximum and starts to decrease. At this point, the course of the patient will be determined by the ability of their immune system to control the viral infection.
(3A) Recovery stage. If the immune response of the patient is able to control infection and the viral load progressively decreases, inflammatory markers decrease and lymphocyte populations recover [12].
(3B) Severe stage. If the immune system fails to control infection, the lymphocyte populations further decrease, whereas viral load and inflammation biomarkers keep increasing. The status of the patients progressively worsens and may lead to death [12].

Although the underlying mechanisms of this inflammatory behavior are scarcely understood, some of the cellular processes identified may help us better understand the course of the disease.

Concerning initial response to the virus, low or delayed IFN-I expression has been associated with a poorer prognosis [10]. The virus has been proven to reduce *IFN* *-β* and *IFN-* *β* gene expression in monocytes during infection [13]. This involves a reduction of antiviral response associated with these molecules and the expression of other molecules such as those of the major histocompatibility complex (MHC) class I, cathepsins, lysosomal and proteasome proteins, all involved in antigen presentation and type 1 (antiviral) immune response [13], [14].

Some COVID-19 patients exhibit a massive release of cytokines called “cytokine storm”, a process that mimics other processes in other diseases such as SHLH. Although the causes that induce this release are unknown, the primary block of IFN-I has been suggested to induce an exaggerated secondary response in other cytokines [15]. Elevated levels of cytokines such as IL-6, TNF-⍺, IFN-γ or IL-8 [15], [16] are observed. One of the main proinflammatory cytokines, IL-1β, it is not increased in peripheral blood of these patients, as it has a short half-life and degrades rapidly. This aspect should be considered when monitoring patients receiving biological therapies, as IL-1β is not a useful parameter for patient monitoring [17].

About lymphocyte subpopulations, the few studies published so far reveal that T CD4^+^ and CD8^+^ cells are the most notably decreased populations, followed by NK and B cells. This decrease is more significant in critical patients and patients with higher levels of proinflammatory cytokines. A reduction has also been observed in regulatory T lymphocytes [18].

On the acute stage, patients with an adequate specific immune response, both humoral and cellular, start to control the virus [19]. Increases in the percentages of circulating antibody-secreting plasmablasts (CD19^+^ (CD19^+^CD27^high^CD38^high^)) and circulating follicular helper T lymphocytes (follicular helper -Tfh-)(CD4^+^CXCR5^+^ICOS^+^PD-1^+^) have been reported from day 7 after the onset of clinical symptoms [20]. An increase has also been observed in T CD8^+^ cells with activated phenotype (CD38^+^HLA-DR^+^) in patients with a favorable course and with good response to immunomodulatory treatments [21]. This increase is not observed in critical patients, who exhibit higher levels of T CD8^+^ cells with exhausted phenotype (PD-1^+^ CTLA-4^+^ TIGIT^+^) [22].

The cellular response that develops is fundamentally a type-1 immune response. Studies in patients infected by SARS-CoV have demonstrated the presence of specific IFN-γ secreting T CD4^+^ and CD8^+^ cells against viral antigens [23]. T CD4^+^ cell populations against SARS-CoV have been identified in the airways even 11 years after infection [24]. These cell populations react rapidly against the virus by secreting IFN-γ and promoting the homing of pulmonary dendritic cells loaded with the antigen to mediastinal lymphatic nodes and attracting specific T CD8^+^ cells to the lungs [23], [24], [25]. Further studies of minor lymphocyte subpopulations in peripheral blood are needed to monitor these changes throughout the course of the disease.

### Humoral response

As this is a novel coronavirus, little is known about humoral response in infected patients. In this setting, it is important to identify seroconversion kinetics, antibody titers and their duration in plasma once the infection has been resolved, as well as their ability as neutralizing antibodies.

Therefore, some authors have focused on humoral response in other similar viruses such as SARS-CoV. Li et al. [26] analyzed humoral immune response in 20 patients to observe the kinetics of immunoglobulins from the onset of the disease until week 12 [26]. Patients were IgG-positive from week 3 until up to 3 months from the onset of symptoms. These antibodies primarily recognized proteins S and N of the virus [27]. On the other hand, IgM antibodies appeared during the first days and were detectable until week 12. Given these results, the authors suggest that IgG antibodies against SARS-CoV play a major role in protection against SARS infection [27].

More recent studies on the novel SARS-COV-2 showed similar kinetics, although they involved a diversity of antigens and patients.

**Figure 2:**
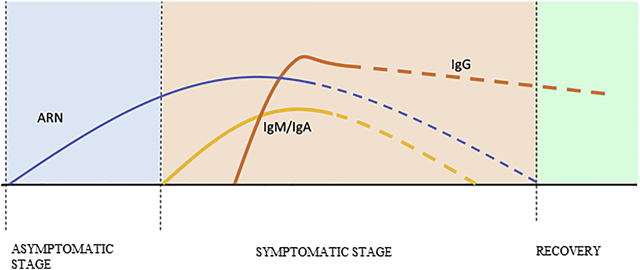
Kinetics of ARN and anti SARS-CoV-2 IgG, IgA and IgM antibodies across the different clinical stages of COVID19.

Guo et al. [28] detected IgM and IgA antibodies already 5 days after the onset of symptoms and continued to be detectable even three weeks later. In addition, IgG antibodies were detected about 14 days after the onset. However, some authors state that IgG antibodies could be detected before, with their plateau occurring at 21 days, with variability among patients [28], [29] (Figure 2).

These results are similar to those reported by other authors as Theravajan et al., who analyzed the kinetics and immunologic response in an Australian patient with SARS-COV-2 [20].

Authors such as Zhao et al. [30] observed the kinetics of the appearance of total antibodies, IgM and IgG at 11, 12 and 14 days respectively, with seropositivity of 50% at day 11 and 100% at day 39 [30].

The appearance of elevated IgG titers before day 14 of onset has been associated with illness severity [30], [31]. The real cause of this effect is unknown, although it is postulated that elevated levels of antibodies induce an increase of immune complexes in lung tissue, which in turn activate the complement, causing inflammation and tissue damage [32]. The presence of antibodies involved in antibody-dependent enhancement has also been reported in SARS-CoV. [33]. The virus-IgG immune complexes attach to *Fc gamma receptors* (FcγR). FcγRs are found in the surface of monocytes, macrophages and B lymphocytes. Fc- FcγR binding would facilitates the entry of the virus into these cells. This infection would not induce a functional viral replication but would induce the activation of infected cells and the generation of proinflammatory cytokines such as TNF-α and IL-6, thereby promoting inflammation [34].

## Diagnostic laboratory techniques

With the emergence of COVID-19 cases, diagnostic laboratory techniques were developed for the early detection and possible immune staging of infected patients.

A diversity of laboratory techniques has been developed for the diagnosis of COVID-19:

(1) Nucleic acid detection techniques: The gold-standard diagnostic technique is based on the so-called RT-PCR or reverse transcriptase polymerase chain reaction, which involves the amplification of the genome of the virus. For this test, to have high sensitivity, it is recommended to collect upper and lower respiratory samples [35]. The advantages of this technique are its specificity and sensitivity (especially within the first 7 days from the onset of symptoms), and the large number of samples that can be simultaneously processed. False negative results can be obtained [36], [37] due to pre-analytical errors (inadequate sampling, poor storage conditions) [38] or due to a low viral load in the sample [39]. From day 14 of the onset of symptoms, the sensitivity of RT-PCR decreases below 50%. Other complementary techniques have been proposed such as antibody determination for the classification of patients [28].
(2) Antigen detection techniques: This technique is based on the detection of the specific antigens of the virus (generally, protein S or N). However, there is limited evidence on the sensitivity and specificity of these tests. The study undertaken by Diao et al. [40] showed a sensitivity of 68% and a specificity of 100% in samples of patients with a positive RT-PCR result with Ct (threshold cycle) <40, whereas sensitivity and specificity increased to 98 and 100%, respectively, when Ct was <30 [40]. The main advantage of this technique is the turnover time (15–30 min), as it involves an immunochromatography assay (rapid tests, also known as Point Of Care). Nevertheless, its usefulness in routine practice is controversial.
(3) Antibody detection techniques: This technique involves the detection of IgA, IgM or IgG antibodies in COVID-19 patients. There are semiquantitative (ELISA or CLIA assays) or qualitative (immunochromatography (lateral flow), point of care) detection techniques. As immune response is detectable 7–11 days after exposure to the virus, (although some patients may develop antibodies before) [38]. This test should not be performed during the first week from the onset of symptoms, as it has a low sensitivity. However, from day 7, the combination of RT-PCR and antibody detection will have a diagnostic sensitivity of 95% [28]. From day 7 of onset of symptoms, the sensitivity and specificity of these immunochromatography assays can reach 88 and 90.6%, respectively [41]. Consistent results were obtained in ELISA studies [42]. Nevertheless, further studies on antigen detection techniques are needed to determine the sensitivity and specificity of the different techniques [42].

The combination of RT-PCR and specific-antigen detection increases detection to 100% from 15 days of the onset of symptoms [28], [29].

The advantages and disadvantages of each laboratory technique are summarized in Table 1.

**Table 1:** Advantages and disadvantages of the available laboratory techniques for the diagnosis of SARS-CoV-2 infection.

	Advantages	Disadvantages
RT-PCR	– High number of samples can be processed – High specificity – High sensitivity the first 7 days from onset of symptoms	– Difficult sampling (nasopharyngeal) – Long turnover time
Viral antigen	– Rapid	– Only a small amount of samples can be tested simultaneously – Low sensitivity and specificity – A minimum detectable antigenic load is required
Antibodies	– Rapid – Good sensitivity from day 7 of onset of symptoms – High specificity – From day 7, antibodies combined with RT-PCR provide a high diagnostic sensitivity – Samples to be tested (serum, plasma and peripheral blood)	– IgA, IgM and IgG kinetics vary across patients

RT-PCR, polymerase chain reaction.

### Relevance of antibody detection

The determination of antibodies as a complementary test to RT-PCR or other techniques can be useful to confirm the diagnosis and reduce the percentage of false negative results, thereby increasing diagnostic sensitivity [28]. These tests also help in disease staging (Table 2) [44].

**Table 2:** Interpretation of RT-PCR results combined with IgA/IgM and IgG antibodies in SARS-CoV-2 infection. Adapted from [43].

Result			Clinical interpretation
PCR	IgA/IgM	IgG	
**−**	**−**	**−**	Negative
**+**	**−**	**−**	Window period
**+**	**+**	**−**	Early stage of infection
**+**	**+**	**+**	Active stage of infection
**+**	**−**	**+**	Final stage of infection
**−**	**+**	**−**	Early stage with false negative. Confirm result with PCR
**−**	**+**	**+**	Disease in progress Confirm result with PCR
**−**	**−**	**+**	Convalescence

PCR, polymerase chain reaction.

Based on the evidence provided above and the document proposed by the *Spanish Society of Immunolog*y [44], the detection of anti-SARS-CoV-2 antibodies is also useful in patients with symptoms of COVID-19 and negative RT-PCR results.

There is little evidence on the protective immunity that patients can develop against SARS-COV-2. Only a study has been published, where immunity was assessed in monkey Rhesus [45]. In this study, the authors observed that the monkeys that generate high levels of neutralizing antibodies in the early stage of the disease are not re-infected later. Although further studies are needed, these results support the usefulness of testing for antibodies in the general population to detect asymptomatic patients and those who developed protective immunity [44].

### Reagents available in the market for the detection of antigens and antibodies

A large variety of reagents are currently available for the detection of antigens and anti-SARS-CoV-2 antibodies, many of which are in validation process or CE-certified. A thorough comparative study is available on the repository “Foundation for Innovative New Diagnostics (FIND)” [46]*.*

However, validation is recommended prior to their incorporation to routine laboratory practice. In addition, due to the high demand during the pandemic, the availability and delivery time must be verified before a reagent is selected.

In a recent study led by Lassaunière et al, a range of serological tests (two ELISA and six immunochromatography assays Point of Care) of different manufacturers were analyzed. The specificity and sensitivity of each test were assessed, and a comparison was performed of methods and internal validations to facilitate choice [47]. The “COVID-19 testing project” was launched in USA to assess and compare the characteristics of reagents available in the market [48].

Chemiluminescence immunoassays (CLIA) on automated platforms are also in progress. Few studies use this technique as a diagnostic tool. Lin et al [49], in a comparative study of ELISA vs. CLIA, reported that CLIA had a higher sensitivity and specificity.

Although COVID-19 is a new disease, validation prior to incorporation to practice is important. Validation allows assessing potential cross-reactivity with other coronaviruses and obtaining an optimal diagnostic result and follow-up of the disease.

## Therapeutic management of COVID-19

Two months after the outbreak of the pandemic in our country, no therapeutic approach has yet been established. A large number of clinical trials are in progress to identify/reposition COVID-19 therapies, and the therapeutic spectrum is changing constantly.

There are two large groups of drugs for COVID-19: those intended to stop viral replication and those aimed to reduce systemic hyperinflammation [50].

The most widely studied drugs that block viral replication are:

– Lopinavir/Ritonavir: Lopinavir is an inhibitor of HIV protease that has been extensively used by health authorities in China for the treatment of COVID-19 [51].
– Remdesivir: It is a nucleotide analogue that interferes with viral RNA polymerization. Although some studies attribute a clinical benefit to this agent, further studies are necessary for a generalized use of this agent in COVID-19 patients [52].
– Hydroxychloroquine/chloroquine: These antimalarial drugs have demonstrated to have antiviral capacity against SARS-COV-2 *in vitro.* They have been proven to interfere with the entry and replication of the virus. These agents also interfere with TLR (toll like receptors) signaling, thereby reducing the activation of immunity [53]. Nevertheless, their clinical benefit is still controversial [54].

To successfully reduce hyperinflammation in this disease, it is important to consider how initial immune response to any antigen works. In this response, macrophages, granulocytes, dendritic cells, epithelial and endothelial cells detect the presence of proteins and RNA/DNA of the pathogen through TLR and NLR-type receptors (NOD like receptors), among other receptors. The activation of these receptors induces the release of IL-1β and IL-6. These cytokines trigger an inflammatory cascade that involves the production of other cytokines and chemokines, causes fever, the production of acute-phase reactants in the liver and the generation of leucocytes in the bone marrow [55], [56].

Thus, different agents with varying specificity can be used to reduce hyperinflammation:

– Corticosteroids: These drugs have been extensively used in COVID-19 patients. However, its effectiveness has not been clearly demonstrated. Thus, a range of studies shows that these agents can exert deleterious effects on patients, although opposite conclusions have been drawn in other studies [54], [57]. The Spanish Agency for Medicines and Medical Devices (AEMPS) do not recommend their use or limit it to very specific settings [57], [58]
– JAK inhibitors [Jakinibis]: JAK [Janus Kinase] inhibitors are drugs that block the transmission of the activation signal of receptors of numerous cytokines (IL-6, IL-4, IL-17, IL-12, IL-10, etc.) and the activation of STAT transcription factors. With these agents, the effect of the cytokine cascade is inhibited at different levels [59].
– Monoclonal antibodies: Based on the key role of cytokines IL-1 and IL-6 in the inflammatory cascade, some antibodies have been tested to try to block these cytokines in COVID-19 patients:

Tocilizumab and sarilumab. Tocilizumab is an antibody anti-IL6 receptor that inhibits the binding of IL-6 to its receptor, thereby blocking its action on the target cell. Sarilumab is an IL-6 inhibitor. AEMPS recommends the use of these agents when IL-6 levels exceed 40 pg/mL [58], [60].

Anakinra. It is a recombinant version of a physiological antagonist of IL-1 receptor [61].

Other potential strategies for the treatment of COVID-19 include the use of hyperimmune plasma of patients who have recovered from the infection [62] or treatment with blocking monoclonal antibodies [63]. All these treatments are under study and there is no robust evidence available on their effectiveness [54].

As mentioned above, the effectiveness of the different therapies used against COVID-19 are continuously revised and updated by the AEMPS [64].

## The search for a vaccine: past, present and future

Since the outbreak of SARS-COV-2, numerous research studies and clinical trials have been undertaken to develop safe and effective vaccines against the virus [65].

A variety of potential vaccines are being tested based on the experience with other coronaviruses such as SARS and MERS [66]. Strategies include the use of whole proteins of the virus, viral peptides, messenger RNA (mRNA) or the inactivated virus.

It is crucial to find the adequate antigen that generates protective, safe and long-lasting immunity. In this sense, protein S (spike) of the virus has demonstrated to have a higher potential to trigger an effective humoral and cellular response [66]. The formation of neutralizing antibodies against this antigen would involve blocking the binding of the virus to the ACE2 receptor and subsequent infection of the target cell [67].

Vaccines based on viral vectors offer a high level of protein expression and long-term stability and induce strong immune responses. The use of adjuvants could improve immunogenicity [68] and perpetuate humoral response [69]. The generation of memory cellular immune response should also be considered for the development of a vaccine [69].

At present, there are more than 90 ongoing projects for the development of a vaccine against COVID-19, of which some already are in initial clinical phases [69], [70]. At this moment, the international scientific community is making considerable efforts to develop effective therapies and vaccines against novel SARS-COV-2. The world is expectant to see how these projects will develop and to what extent these therapies can be applied to the population.
